# Optimizing Agronomic, Environmental, Health and Economic Performances in Summer Maize Production through Fertilizer Nitrogen Management Strategies

**DOI:** 10.3390/plants12071490

**Published:** 2023-03-29

**Authors:** Ling Zhang, Wu-Shuai Zhang, Qing-Feng Meng, Yun-Cai Hu, Urs Schmidhalter, Cheng-Hu Zhong, Guo-Yuan Zou, Xin-Ping Chen

**Affiliations:** 1 College of Resources and Environment, Academy of Agricultural Science, Interdisciplinary Research Center for Agriculture Green Development in Yangtze River Basin, Southwest University, Chongqing 400716, China; 18854806552@163.com (L.Z.); wszhang@swu.edu.cn (W.-S.Z.); 2 Institute of Plant Nutrition, Resources and Environment, Beijing Academy of Agriculture and Forestry Sciences, Beijing 100097, China; gyzou@163.com; 3 Department of Plant Sciences, Chair of Plant Nutrition, Technical University of Munich, 85354 Freising, Germany; hu@wzw.tum.de (Y.-C.H.); schmidhalter@wzw.tum.de (U.S.); 4 College of Agronomy and Biotechnology, China Agricultural University, Beijing 100193, China; mengqf@cau.edu.cn; 5 Moith Agricultural Technology Co., Ltd., Chizhou 242800, China; zhongch@moith.com

**Keywords:** nitrogen, maize (*Zea mays* L.), grain yield and quality, environmental impacts, human health impact, economic analysis

## Abstract

Although nitrogen (N) fertilizer application plays an essential role in improving crop productivity, an inappropriate management can result in negative impacts on environment and human health. To break this dilemma, a 12-year field experiment (2008–2019) with five N application rates was conducted on the North China Plain (NCP) to evaluate the integrated impacts of optimizing N management (Opt. N, 160 kg N ha^−1^ on average) on agronomic, environmental, health, and economic performances of summer maize production. Over the 12-year study, the Opt. N treatment achieved the maximal average grain yield (10.6 Mg ha^−1^) and grain protein yield (793 kg ha^−1^) among five N treatments. The life cycle assessment methodology was applied to determine the negative impacts on environmental and human health, and both of them increased with the N rate. Compared with the farmers’ conventional N rate (250 kg N ha^−1^), the Opt. N treatment reduced acidification, eutrophication, global warming, and energy depletion potentials by 29%, 42%, 35%, and 18%, respectively, and reduced the health impact by 32% per Mg of grain yield or grain protein yield produced. Both the Opt. N and Opt. N*50–70% treatments resulted in high private profitability (2038 USD ha^−1^), ecosystem economic benefit (1811 USD ha^−1^), and integrated compensation benefit (17,548 USD ha^−1^). This study demonstrates the potential benefits of long-term optimizing of N management to maintain high maize yields and grain quality, to reduce various environmental impacts and health impacts, and to enhance economic benefits. These benefits can be further enhanced when Opt. N was combined with advanced agronomic management practices. The results also suggest that reducing the optimal N rate from 160 to 145 kg N ha^−1^ is achievable to further reduce the negative impacts while maintaining high crop productivity. In conclusion, optimizing the N management is essential to promote sustainable summer maize production on the NCP.

## 1. Introduction

Maize is one of the most important cereal crops and it provides about 20% of the calories and 15% of the proteins for the global food diet [1]. In China, more than 30% of maize grains were produced on the North China Plain (NCP), mainly as summer maize [2]. Due to the important role of N as an essential nutrient for crop growth, the local farmers usually apply excessive N fertilizer rates as an ‘insurance’ to pursue high maize yield [3]. The overuse of N fertilizer, however, did not further increase maize yield, but resulted in high risks of N lost to the environment [3], which also brings risks to human health [3,4,5,6,7]. For a sustainable agricultural production system, it is urgently needed to optimize N management to balance the trade-offs between maize production and environmental costs.

Previous studies have shown that optimizing N fertilizer rates in maize production systems can maintain high yield while mitigating the global warming potential (GWP) from greenhouse gases emissions [3,7,8,9]. However, most of these studies evaluated the impact of N management practices on a time scale within only a few years, which may be highly affected by inter-annual climate variability. Therefore, further studies are needed to quantify the long-term sustainability of N management. In addition to GWP, the soil acidification potential (AP), eutrophication potential (EP), and energy depletion (ED) potential were also largely dependent on the N management [10], whereas the effects of the N management on these environmental impacts remains unclear. Moreover, maize is primarily used as an animal feed [11,12], and the grain protein content is considered an important nutritional quality indicator [13]. In most previous studies, the environmental burdens of maize production were assessed and expressed on a land area- or crop yield-base [9,14]. However, it is increasingly recognized that the nutritional quality (i.e., grain protein yield) also needs to be considered to better understand the balance between environment and production. More studies and modelling approaches are needed to evaluate the long-term effects of N management on grain yield and quality [13], and broad-range environmental impacts of summer maize production systems [10].

In addition to food security and environmental quality [15], guaranteeing human health and maximizing economic benefits are also sustainable development goals [16,17]. Concentrations of air pollutants, such as ammonia (NH_3_), nitrogen dioxide (NO_2_), sulfur dioxide (SO_2_), were found to be positively related to the mortality and morbidity rates of respiratory and cardiopulmonary diseases in humans [18,19,20,21,22]. Recently, more studies have focused on the human health impacts caused by crop production systems. For example, Wang and Lu [23] reported that improving N fertilizer management has a great potential to reduce human health risks in cereals production systems in China. In addition, several studies have assessed the life-cycle health impacts and the induced human capital losses (HCL) in the traditional farmers’ maize production system on the NCP [24,25]. However, how and to what extent human health risks could be alleviated through optimizing N management in summer maize production systems remains unclear. Life cycle assessment (LCA) could not only evaluate the potential environmental impacts [26], but also be able to link life-cycle pollutants and health impacts with agricultural production [20].

For crop production, the farmer’s private profitability considering the product incomes and costs of agricultural material inputs is the most direct representation of the applicability of specific N management practice. Furthermore, adverse impacts of N fertilizer application can cause considerable economic costs for ecological restoration and human illness [4,27], which also should be considered for evaluating the ecosystem economic benefit (EEB) to reflect he monetary value for public interests [28,29]. Wang and Zhao [25] reported that the health-induced HCL approach could be used to formulate environmentally friendly policies regarding ecological compensation for crop production. Therefore, the quantitative economic evaluations considering costs from multiple perspectives (ecosystem cost, health cost, and HCL) can provide more comprehensive policy guidance for sustainable agricultural production systems.

The specific objectives of this study were: (1) to determine the impacts of long-term N management on yield and quality of maize grain; (2) to quantify the various environmental impacts (AP, EP, GWP, and ED) and human health impacts induced by N management and identify their main contribution factors; and (3) to evaluate the multiple economic benefits of optimizing N management over the long run. This research was based on a consecutive 12-year summer maize field experiment with five N treatments conducted on the NCP. The novelty is to determine whether there are trade-off relationships among agronomic, environmental, health, and economic performances in the maize production system and how to optimize the fertilizer N practice to manage the trade-offs. The study also evaluates the sustainability of summer maize production through N management from multiple dimensions, to provide a more comprehensive view for the policy-makers and stakeholders.

## 2. Results and Discussion

### 2.1. Grain Yield and Grain Protein Yield 

**Figure 1:**
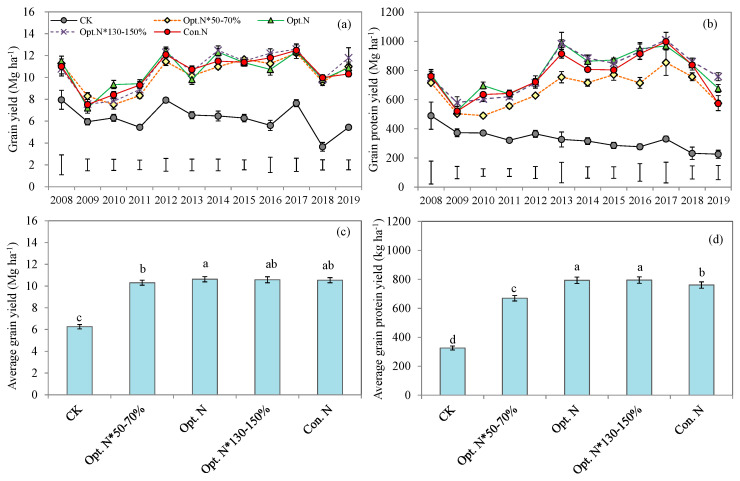
Annual grain yield (**a**), annual grain protein yield (**b**), average grain yield (**c**), and average grain protein yield (**d**) of summer maize under five N treatments during the years 2008–2019 of the experiment. CK, no N; Opt. N*50–70%, 50–70% of optimal N rate; Opt. N, optimal N rate; Opt. N*130–150%, 130–150% of optimal N rate; Con. N, conventional N rate. Bars are LSD at *p* < 0.05. Means followed by the same lowercase letter are not significantly different among the five N treatments at *p* < 0.05 according to LSD. Vertical bars represent means of 12 growing seasons ± SE (*n* = 48).

Across the 12 cropping seasons, the mean maize grain yield and grain protein yield increased with the increasing N rates from CK to the Opt. N treatment (160 kg N ha^−1^ on average), whereas more N inputs exceeding the Opt. N level did not show further improvement (Figure 1, Tables S2 and S4). Compared to CK treatment, the Opt. N treatment significantly increased the mean grain yield by 70% (10.6 vs. 6.3 Mg ha^−1^) and grain protein yield by 143% (793 vs. 326 kg ha^−1^). Compared to the Opt. N *50–70%, the Opt. N treatment improved grain yield by 3% (10.6 vs. 10.3 Mg ha^−1^) and grain protein yield by 18% (793 vs. 670 kg ha^−1^) (Figure 1). Since the integrated agronomic management practices were employed (especially increased plant density and modified seeding date) in 2012, higher grain yield and grain protein yield were achieved than before (Table S5). Under the Opt. N treatment, the mean grain yield raised from 9.4 Mg ha^−1^ during 2008–2011 to 11.3 Mg ha^−1^ during 2012–2019, and the grain protein yield increased from 658 to 860 kg ha^−1^ (Table S5). The high yield of 11.3 Mg ha^−1^ reached 85% of the yield potential on the NCP (13.3 Mg ha^−1^) and was 37.8% greater than the farmers’ average yield in China (8.2 Mg ha^−1^) [30]. The results showed that, under the current climate and soil production conditions, Opt. N treatment had achieved a quiet high yield level.

**Figure 2:**
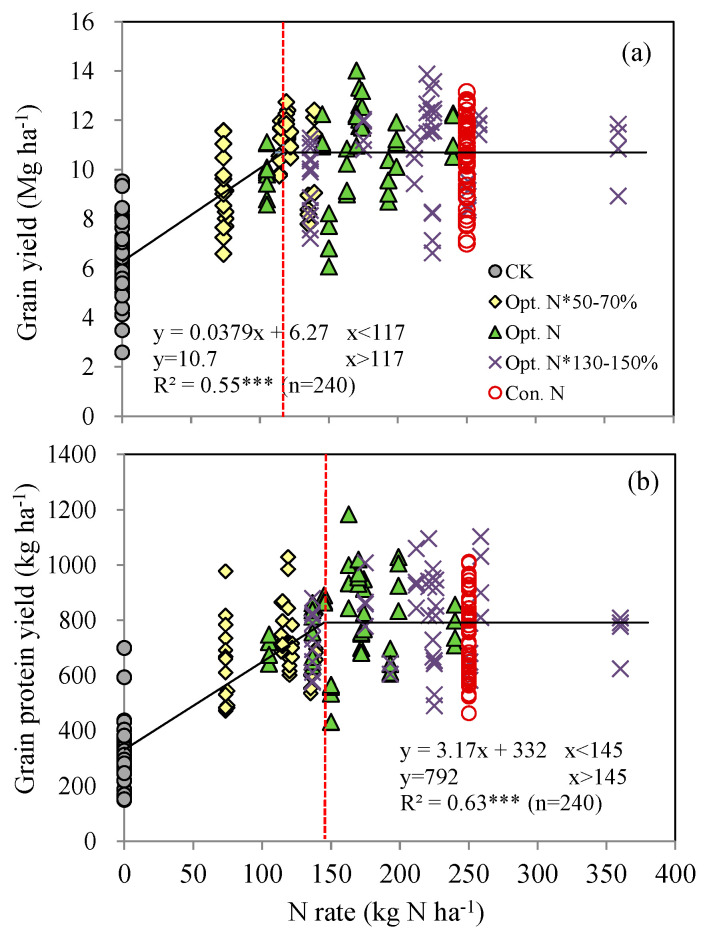
The relationships between grain yield (**a**) or grain protein yield (**b**) and N fertilizer application rate in summer maize production system during the years 2008 to 2019 of the experiment. *** represents significant difference at *p* < 0.001.

The relationship between grain yield and N rate was best fitted by a linear–plateau model, and the same trend was also observed between grain protein yield and N rate (Figure 2). The critical N rates for maximal yield and protein yield were 117 and 145 kg N ha^−1^, respectively. The corresponding maximal yield and protein yield was 10.7 Mg ha^−1^ and 792 kg ha^−1^, respectively, with both being close to the actual values obtained in the Opt. N treatment (Figure 1 and Figure 2). Such results suggest that the Opt. N treatment has achieved the best maize production and grain quality. Under the Opt. N treatment, fertilizers were split into three applications by fully considering the various environmental N resources and inherent soil inorganic N, thus synchronizing N supply with crop demand temporally and spatially [28]. Therefore, even though the application rate in the Opt. N treatment was 36% lower than that in the Con. N treatment, it had reached the maximal yields and grain quality. Figure 2 shows that more N fertilizers would be needed to achieve the maximal grain protein yield than the grain yield, likely due to the higher energy requirements for the synthesis of protein compared to carbohydrate [31]. In addition, the current results also indicated that there is still potential for further lowering the optimal N rate, because the 145 kg ha^−1^ N fertilizer input was enough to guarantee the maximal yield and grain protein yield. 

### 2.2. Environmental Impacts and Resource Consumption 

**Figure 3:**
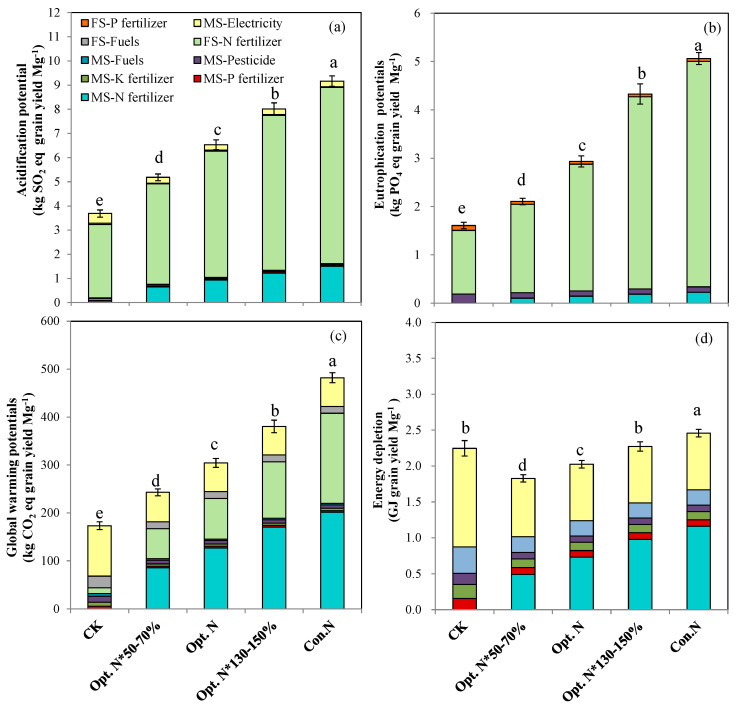
Average life-cycle acidification potential (**a**), eutrophication potential (**b**), global warming potential (**c**), and energy depletion potential (**d**) per Mg of maize grain produced under five N treatments during the years 2008–2019 of the experiment. CK, no N; Opt. N*50–70%, 50–70% of optimal N rate; Opt. N, optimal N rate; Opt. N*130–150%, 130–150% of optimal N rate; Con. N, conventional N rate. MS, agricultural materials system; FS, arable farming system. Means followed by the same lowercase letter are not significantly different among five N treatments at *p* < 0.05 according to LSD. Vertical bars represent means of 12 growing seasons ± SE (*n* = 48).

**Figure 4:**
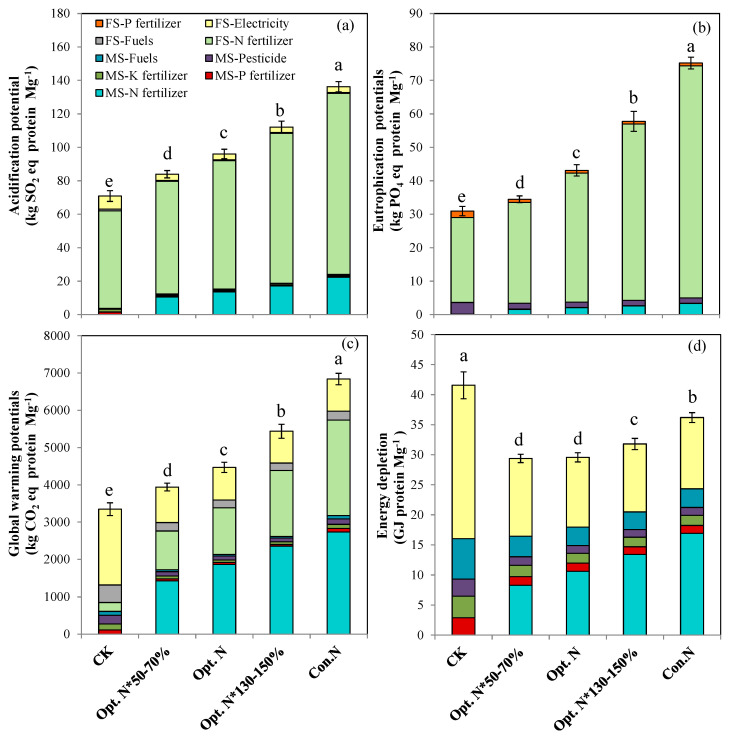
Average life-cycle acidification potential (**a**), eutrophication potential (**b**), global warming potential (**c**), and energy depletion potential (**d**) per Mg of maize grain protein produced under five N treatments during the years 2008–2019. CK, no N; Opt. N*50–70%, 50–70% of optimal N rate; Opt. N, optimal N rate; Opt. N*130–150%, 130–150% of optimal N rate; Con. N, conventional N rate. MS, agricultural materials system; FS, arable farming system. Means followed by the same lowercase letter are not significantly different among five N treatments at *p* < 0.05 according to LSD. Vertical bars represent means of 12 growing seasons ± SE (*n* = 48).

Over the entire crop production cycle, the AP, EP, and GWP based on grain yield and grain protein yield showed the same trend, being that all three increased with increasing N application rates (Figure 3 and Figure 4). In contrast, the ED decreased first and then increased with increasing N rates. The lowest ED occurred with the Opt. N*50–70% treatment when expressed as per Mg of grain produced (Figure 3), and occurred with the Opt. N*50–70% and Opt. N treatments when expressed as per Mg of grain protein produced (Figure 4). Compared with the Con. N treatment, the Opt. N reduced the AP, EP, GWP, ED by 29%, 42%, 37%, and 18% when expressed on grain yield (Figure 3), and by 30%, 43%, 35%, and 18% when expressed on protein yield, respectively (Figure 4). 

Under the Opt. N treatment, the mean GWP, AP, EP, and ED per Mg of grain produced were 304 kg CO_2_ eq, 6.5 kg SO_2_ eq, 2.9 kg PO_4_ eq, and 2.0 GJ, respectively (Figure 3, Table S6). These environmental impacts were lower than values reported in previous studies in the double high cropping system or on a survey of farming practices conducted on the NCP (Table S6) [10,24,32,33]. Across a broad range of the USA Corn Belt, the environmental impacts were comparable to the results of the present study (Table S6) [34]. These comparison results suggested that optimizing N management for maize production on the NCP showed favorable environmental performance, mainly due to the lower N fertilizer inputs and higher grain yield in the improved production system.

Additionally, the key contributors to each environmental impact category were identified on both yield and protein yield production basis. The on-field N fertilizer application was the most contributing factor to AP and EP in the N fertilized treatments, with the proportion of 80% for AP and 87–92% for EP. This is attributed to that NH_3_ volatilization derived from N-fertilizer-application-dominated AP, and the on-field leaching losses of NO_3_^−^ and NH_3_ dominated EP (Figure 3 and Figure 4). Furthermore, pollutant emissions during the manufacture and transport of N fertilizers were the secondary contributors (SO_2_ and NO_x_ contributed to AP; NH_4_ and NH_3_ contributed to EP), and the contribution of other materials was negligible. For GWP, the manufacture and transport of N fertilizer and on-farm nitrous oxide (N_2_O) emissions contributed with a share of 36–43% and 26–43%, respectively (Figure 3 and Figure 4). In addition, electricity generation used for irrigation was identified as a significant contributing factor, accounting for 12–24% of the total GWP under N application treatments. In contrast, the manufacture and transport of N fertilizer and electricity generation had a comparable contribution to ED, with the share ranging 28–47% and 32–44%, respectively. In general, N fertilizer was the largest contributing factor to AP, EP, GWP, and ED.

By analyzing the contribution sources of environmental impacts, the main reasons for Opt. N to reduce environmental impacts compared with Con. N was revealed, which could be attributed to two factors. First, on the arable farming stage, the on-field NH_3_, NO_3_^−^, and N_2_O losses resulted in soil acidification, water eutrophication, and global warming. Previous studies on the NCP found that NH_3_ volatilization increased linearly with N rates. In addition, NO_3_^−^ leaching and N_2_O emissions increased exponentially with N rates [8,35]. Therefore, the reduced N rates in the Opt. N treatment substantially decreased the various N losses, thereby mitigating the negative environmental impacts. Second, lower pollutants (SO_2_, NO_x_, NH_4_, and NH_3_) were generated and lower energy was consumed during the N fertilizer production and transport process due to a lower N rate in the Opt. N treatment, which also reduced various environmental impacts. The results of this study clearly demonstrate the importance of optimizing N inputs in reducing environmental risks. It is also worth noting that the acidification and eutrophication potentials were higher than those in Poland (Table S6). Besides the differences in N rate and grain yield, the higher risks on the NCP are also associated with weather conditions. The concentrated rainfall and high temperatures in summer may lead to more N losses on the NCP [3]. Therefore, in addition to optimizing the N fertilizer rate, other effective approaches, such as the use of controlled-release N fertilizer or urease/nitrification inhibitors, may further reduce N losses and environmental impacts [36,37].

In addition to N rate, yield and protein yield variability also highly affected the environmental performance indirectly. As it can be seen, the negative environmental impacts were reduced in the recent 8 years by implementing an improved agronomic management strategy (Figures S1 and S2), which was mainly attributed to the greater yield and protein yield being achieved. Previous studies also reported that the environmental impacts were affected by yield level [14]. These results suggested that combining the optimal N management with advanced agricultural practices can greatly alleviate the environmental risks. Similarly, Huang et al. [9] also pointed out that an optimized N management in combination with advanced agronomic measures could maintain grain yields while mitigating environmental costs. The year-to-year variations in yield and protein yield were largely dependent on climate differences (Table S4). From this perspective, long-term experiments in this study provide an opportunity in assessing the year-to-year climate variability, and can provide more solid evidence for sustainable N management in the maize production system.

### 2.3. Human Health Impact 

**Figure 5:**
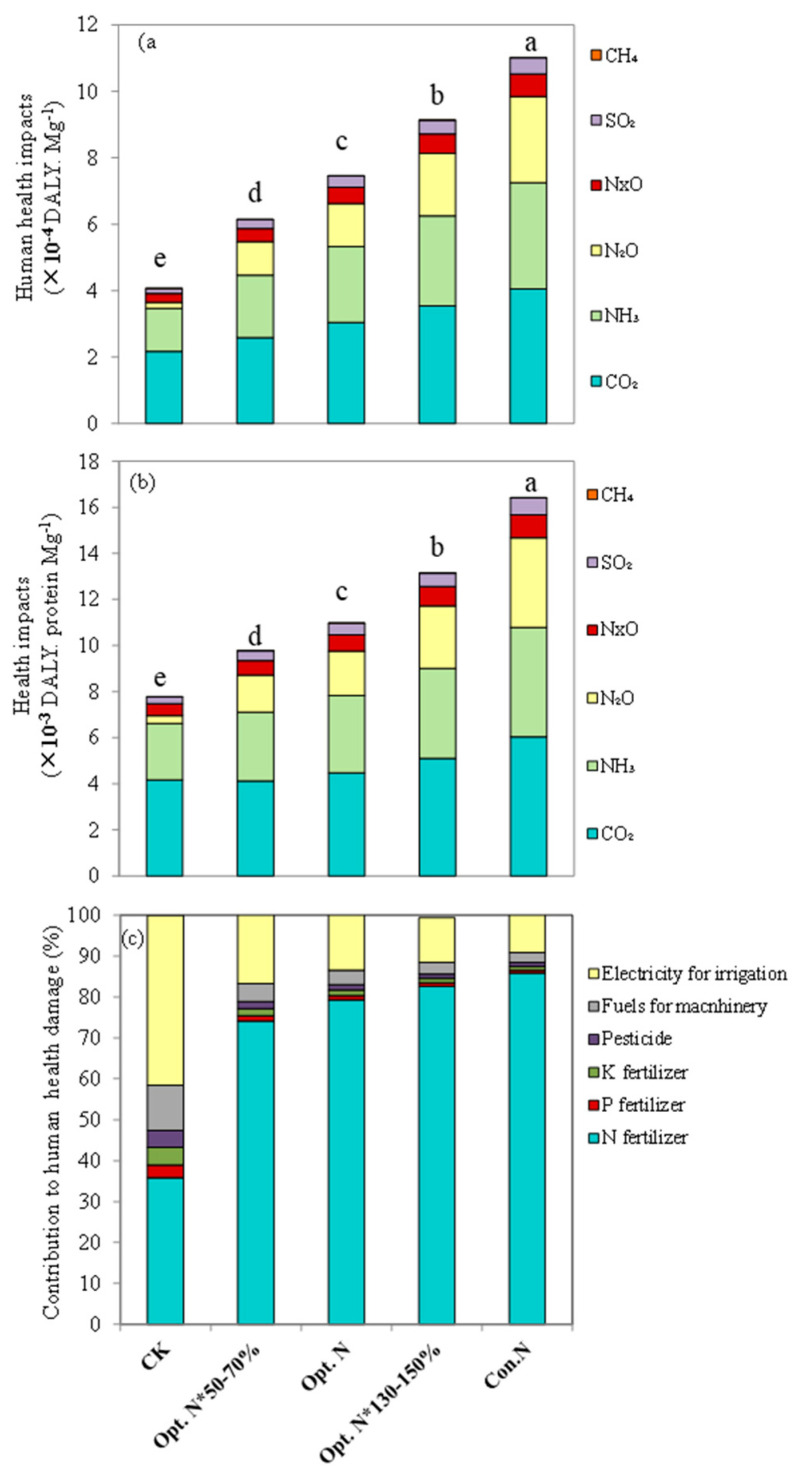
Average human health effects per Mg of maize grain produced (**a**) and per Mg of maize grain protein produced (**b**) and contributions of agricultural materials input to the human health effect (**c**) under five N treatments during the years 2008–2019 of the experiment. CK, no N; Opt. N*50–70%, 50–70% of optimal N rate; Opt. N, optimal N rate; Opt. N*130–150%, 130–150% of optimal N rate; Con. N, conventional N rate. Values are means of 12 growing seasons (*n* = 48). Means followed by the same lowercase letter are not significantly different among five N treat-ments at *p* < 0.05 according to LSD.

Across the entire maize production cycle, the human health impacts caused by the air pollutant emissions were evaluated. On either yield or protein production basis, the negative human health impacts increased with increasing N rate (Figure 5). Under the Opt. N treatment, the human health impacts were 7.45 × 10^−4^ DALY per Mg grain yield produced and 1.10 × 10^−2^ DALY per Mg grain protein yield produced, which declined by 32% and 33% compared with the Con. N treatment (Figure 5). Among all the air pollutants, CO_2_ and NH_3_ contributed the most serious potential damage to human health, with a partial contribution of 37–42% and 30%, respectively, in the N fertilized treatments, followed by N_2_O (16–24%). NO_x_ and SO_2_ contributed a small share to the health impacts, whereas the contribution of CH_4_ was negligible. 

Furthermore, the contribution proportion of different agricultural material inputs to human health impacts was determined. The results showed that, under the N application treatments, 74–85% of the human health impacts were attributed to N fertilizer input, and the contribution share increased with N rates (Figure 5). Electricity generation used for irrigation was the second-largest contributor, accounting for 9–17%. Similarly, Wang and Zhao [25] concluded that N fertilizer was the largest contributor to pollutant-induced human health impacts, followed by electricity. Such results are associated with the sources of air pollutants. Among the five main air pollutants, almost all NH_3_ and N_2_O were emitted from the on-field N fertilizer application. Moreover, most of the CO_2_, NO_x_, and SO_2_ were emitted during the N fertilizer production process, and the remaining small proportion was from the electricity generation process. That is to say, the main air pollutants contributing to human health impacts were closely related to N fertilizer input. In China, the production process of electricity and N fertilizer mainly relies on coal, with high emission density, so the pollutant emissions are relatively high [38]. Therefore, in the agricultural production system, reducing the life-cycle environmental impacts and human health impacts also depends on the improvement of industrial production technology, such as increasing the use of clean energy.

### 2.4. Economic Benefits Analysis

Grain yield benefit increased significantly with the N application rate up to the Opt. N (3294 USD ha^−1^), and then plateaued (Table 1). After accounting for the costs of N fertilizer and other agricultural inputs, the farmers’ private profitability reached the maximum (2038 USD ha^−1^) in the Opt. N 50–70% treatment and remained stable at greater N rates. EEB also increased with N rates up to the Opt. N (1811 USD ha^−1^), and then decreased thereafter due to the higher social cost. When the HCL was taken into consideration, the highest ICB was achieved in the Opt. N*50–70% (1754 USD ha^−1^) and Opt. N (1747 USD ha^−1^) treatments, and then declined. To reflect the eco-efficiency, the social cost–private profitability ratio was applied as a composite indicator to assess the sustainability of agricultural production systems [39]. The lower social cost–private profitability ratio means higher eco-efficiency. In this study, the social cost–private profitability ratio among the CK, the Opt. N*50–70%, and the Opt. N treatments showed no significant difference, and increased significantly at higher N rates. These results suggest that high eco-efficiency could be achieved in the CK, Opt. N*50–70%, and Opt. N treatments, and increasing N inputs over the optimal level resulted in a low eco-efficiency for summer maize production systems on the NCP.

### 2.5. Effect of N Management on the Trade-Offs among Summer Maize Production, Environmental and Health Performances 

**Figure 6:**
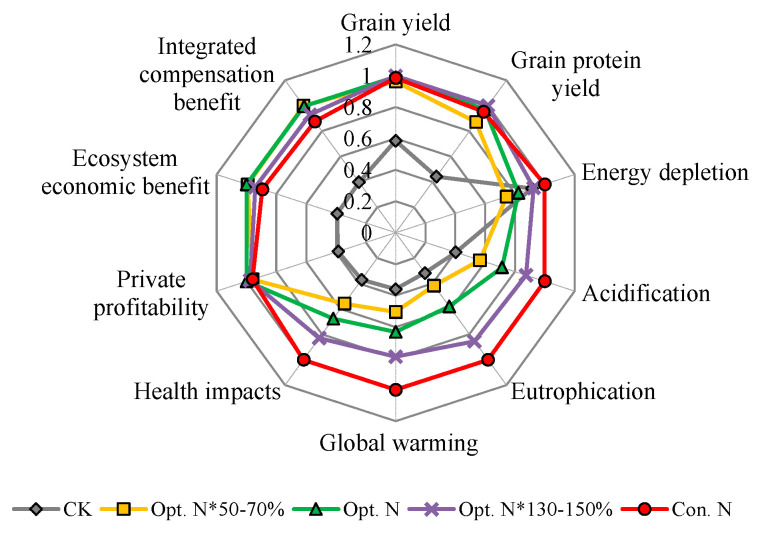
Relationships among maize production, environmental impacts, human health impacts, and economic performance under different N treatments. The environmental impacts include global warming potential, eutrophication potential, acidification potential, and energy depletion potential. The economic performance includes private profitability, ecosystem economic benefit, and integrated compensation benefit. CK, no N; Opt. N*50–70%, 50–70% of optimal N rate; Opt. N, optimal N rate; Opt. N*130–150%, 130–150 % of optimal N rate; Con. N, conventional N rate.

The results of the current study showed the trade-off relationship among productivity, environmental, and health impacts for maize production systems on the NCP. For example, although the environmental and health impacts were lowest in the CK treatment, the grain yield and protein yield were also lowest (Figure 6). The Con. N treatment achieved high maize production, whereas they also substantially increased the environmental and health impacts (Figure 6). Compared to the CK and Con. N treatment, the Opt. N achieved both high maize production and lower environmental and health impacts. It is worth noting that compared to the Opt. N*50–70% treatment, although the Opt. N achieved significantly greater grain and protein yields, the environmental and health impacts were also significantly higher (Figure 6). Under the conditions of the NCP, compared to the fertilized treatments, the maize yield in CK was not very low, which was mainly attributed to the high environmental N inputs (104 kg N ha^−1^ year^−1^) in this region [3]; thus, the N-derived yield and protein yield were not so high. Therefore, the increased grain and protein yields were not great enough to offset the increased environmental and health impacts caused by N fertilizer application. Moreover, the EEB can be used as an effective indicator to balance the trade-offs between productivity and environmental risks, because the EEB considered both the yield incomes and costs of damage to the environment and human health. In addition, the ICB could reflect the trade-offs among productivity, environmental, and human health impacts, because it further considered the human capital loss compensation. In this study, there were no significant differences in the private profitability, EEB, and ICB between the Opt. N*50–70% and Opt. N treatments (Table 1), indicating that both Opt. N*50–70% and Opt. N treatments could maximize farmers’ private profitability and achieve the optimal balance among maize yield production, environmental, and human health impacts (Figure 6). However, when considering the grain quality, the Opt. N*50–70% did not obtain the same grain protein quality as in the Opt. N treatment (Figure 6). 

To meet the global food demands due to the increasing population, ensuring high-yield and high-quality food production is the primary task of agricultural production [40]. In this case, satisfying maize production on the NCP can only be achieved by sacrificing environmental and human health costs to some extent, due to the trade-offs between maize production and environmental and human health impacts (Figure 6). The present study showed that the Opt. N treatment, based on the in-season root zone N management, could ensure high maize yield and high quality with relatively low environmental and health costs. Furthermore, the study shows that it is feasible to further slightly reduce the optimal N rate from 160 kg ha^−1^ to 145 kg ha^−1^ in the long run.

### 2.6. Potential Limitations and Future Applications

This study evaluated whether optimizing N management could achieve the optimal balance among maize productivity, environmental, and human health impacts in the long run. However, there were also some inevitable limitations. First, the reliability of the LCA results is largely dependent on the accuracy of life cycle inventories. For instance, due to the lack of field measurement data, the on-field Nr losses were determined based on the empirical models on the NCP to restrict the uncertainty [8]. Still, there were some uncertainties because Nr losses could vary with regional conditions and management measures, including soil properties, climate, and N applications [41], which were not considered in this study. In addition, the pollutant emissions during agricultural material production and transport processes are another source of uncertainty. In this study, the emission parameters of the agricultural inputs production process were mainly derived from Liang [38], which complied the inventories of usual products in China, and were widely used in the LCA studies [25,42]. However, the accuracy of these inventories needs to be improved due to the incomplete data collection. From the perspective of health impact, the local damage factors of various pollutants are needed to enhance the accuracy of the results. Despite these limitations, this study assessed the integrated effects of long-term optimizing N management on maize production, therefore providing meaningful reference information. The optimized N management in maize production on the NCP could also be implemented to other cropping systems, and could be extended to other regions as well, with careful consideration of crop N demand and site-specific ecological conditions [27].

## 3. Materials and Methods

### 3.1. Experiment Site, Design and Field Management

This 12-year field experiment (2008–2019) was conducted on a calcareous alluvial soil at the Quzhou Experimental Station (36.9° N, 115.0° E), Hebei Province, on the NCP. This region has a warm, sub-humid, continental monsoon climate. The cropping system is dominated by a typical winter wheat—summer maize rotation. The summer maize is generally planted in early June and harvested in early October. In the maize growing seasons during 2008–2019, the mean temperature was 25.3 °C and the mean precipitation was 312 mm.

The field trial followed a randomized complete block design with 4 replicates, and each plot has an area of 20 m long × 15 m wide. The experiment included five N treatments: (1) without N fertilizer as a control (CK), (2) optimal N rate (Opt. N), (3) sub-optimal N rate, Opt. N*50–70% (50% of Opt. N during 2008–2009 and 70% of Opt. N during 2010–2019), (4) supra-optimal N rate, Opt. N*130–150% (150% of Opt. N during 2008–2009 and 130% of Opt. N during 2010–2019), and (5) the farmers’ conventional N rate (Con. N). For the Opt. N, Opt. N*50–70% and Opt. N*130–150% treatments, N fertilizer was spit-applied before planting, at the 6-leaf (V6) stage, and the 10-leaf (V10) stage (during 2008–2011) or silking (R1) stage (during 2012–2019) [43]. The application rates in the optimal N treatments were determined based on an in-season root-zone N management strategy, which considered the N supply from soil, environment, and N fertilizer inputs to match the high-yielding maize variety N requirement in time, space, and quantity [43]. Before planting, 45 kg N ha^−1^ was applied in the Opt. N treatment. The optimal N rate at the V6 and V10 (or R1) growth stage was calculated by subtracting the measured root-zone soil nitrate (NO_3_^−^) content from the corresponding N target values estimated based on the target yields [12]. The N target values are shown in Table S1. For the Con. N treatment, 100 and 150 kg N ha^−1^ fertilizers were applied before planting and at the V6 stage, respectively. Nitrogen was applied as urea throughout the study period and the application rates are listed in Table S2. Phosphorus (P) fertilizer as calcium superphosphate and potassium (K) fertilizer as potassium sulfate were applied before planting. The used maize cultivar was Zhengdan 958, the most popular high-yield hybrid on the NCP.

Since 2012, an integrated soil–crop system management approach was used to maximize crop yield productivity [43]. This approach had improved field management practices for maximum use of fixed resources (water, fertilizer, solar radiation, and favorable growing conditions). The details of agronomic management practices are listed in Table S1. Irrigation was applied before planting and during growing seasons depending on weather conditions. During the 12 maize growing seasons, the total irrigation volume was 85, 50, 70, 140, 90, 75, 90, 170, 90, 170, 90, and 90 mm, respectively. 

### 3.2. Sampling and Laboratory Procedures

At the physiological maturity stage, all maize ears in a center 12 m^2^ (5 m × 2.4 m) area in each plot were harvested and threshed, and the grains were dried and then adjusted to standard grain yield (expressed on a 15.5% humidity basis). Grain N concentration was determined using the Kjeldahl method [44]. Grain protein concentration was calculated by multiplying N concentration by 6.5 [45]. Grain protein yield was calculated as the product of grain yield and protein concentration. 

### 3.3. Life Cycle Assessment (LCA)

#### 3.3.1. Goal, Scope and Functional Unit

**Figure 7:**
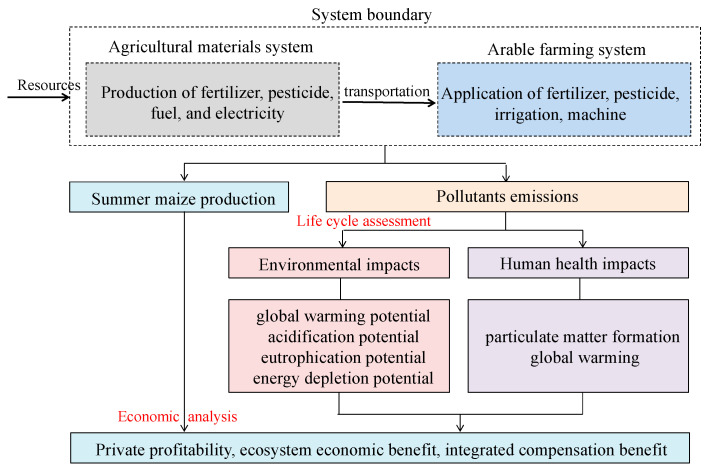
System boundary and analytical framework of using life cycle assessment and economic analysis to evaluate the sustainability of maize production system.

This study aimed to evaluate the potential environmental impacts of summer maize production as affected by different N rates. The system boundary focused on summer maize production that was divided into two parts: the agricultural materials system (MS) (including raw material acquisition, production of agricultural inputs including fertilizer, pesticide, and associated resources of fuel and electricity) and the arable farming system (FS) (Figure 7). The environmental impacts of maize production systems were evaluated based on two function units, i.e., per million grams (Mg) of grain and grain protein.

#### 3.3.2. Life Cycle Inventory Analysis

The LCA inventory in this study considered all the emissions from agricultural materials production and on-field farming processes. For the agricultural materials production and transport, the inventories on the production of N, P, K fertilizers, pesticide, fuel, and electricity were gained from Liang [38] and Yue [46]. In the arable farming processes, on-field N losses were estimated from the site-specific experiments and empirical models on the NCP to restrict the uncertainty [8,35]. The emission factors or equations from fertilizer application are listed in Table S3. The pollution emissions of diesel fuel combustion for agricultural machines were gained from Liang [38]. 

#### 3.3.3. Life Cycle Impact Assessment and Interpretation

Impact assessment analyzes all inputs and outputs inventory data of the maize production system. In this study, four important categories including GWP, AP, EP, and ED, which were affected by N management, were evaluated. According to ISO standards [47,48], for each environmental impact category, the characterization was implemented using the equation:

(1) $$E_{P}=\sum_{i=1}^{n}E_{Fi}\times Rate_{i}$$

where *E_P_* represents each environmental impact potential; *E_Fi_* is the relevant characterization factor of per unit *i* input category or emission, which was adopted from Liang et al. [10]; and *Rate_i_* is the rate of *i* input category or emission. 

Additionally, the endpoint human health impacts caused by the life cycle pollution emissions were evaluated using the Disability Adjusted Life Years (DALYs) method, which represents the potential life years that are lost or associated with a disability. A previous study revealed that almost all human health risks were caused by fine particulate matter formation (induced by NH_3_, NO_x_, and SO_2_) and global warming (induced by CO_2_, N_2_O, and CH_4_) [21]. Thus, the ReCiPe method was used to calculate the potential human health impacts from particulate matter formation and global warming perspectives, using the equation:

(2) $$\mathrm{Human}\;\mathrm{health}\;\mathrm{impacts}=\sum DF_{i}\times Dose_{i}$$

where *DF_i_* is the damage factor of the *i*th pollutant (DALY. kg^−1^), which was derived from the software SimaPro 8.4.0 [25]; *Dose_i_* is the dose of the *i*th pollutant. Figure 7 depicted the analytical framework used in this study.

Based on the potential human health impacts, the human capital loss (HCL) was calculated as:

HCL = Human health impacts × PC_GDP(3)

where PC_GDP represents China’s average per capita GDP during 2008–2019 (USD 7087, https://data.stats.gov.cn, accessed on 1 March 2020).

### 3.4. Economic Benefits Analysis

In this study, three economic indicators, namely farmers’ private profitability, ecosystem economic benefit (EEB), and integrated compensation benefit (ICB), were used to evaluate the monetary value and reflect the producers’ private profitability and public interests (EEB and ICB), respectively. Private profitability considered the grain yield incomes and costs of agricultural materials inputs. In addition to this indicator, EEB also considered the social costs of ecosystem deterioration and health damage, and ICB further accounted for the human capital loss compared to EEB. The farmers’ private profitability, EEB, and ICB were calculated using the equations:

Private profitability = grain yield × W_price_ − N cost − other costs (4)

Ecosystem economic benefit = private profitability − social cost(5)

Integrated compensation benefit = ecosystem economic benefit − human capital loss(6)

where W_price_ represents the market price of maize (averaged 0.32 USD kg^−1^ in local agro-product markets). N fertilizer cost was calculated by multiplying the fertilizer rate by the average fertilizer price (0.62 USD kg^−1^). In addition, other costs (1157 USD ha^−1^ on average over 12 years) considered the costs of P and K fertilizers, pesticides, electricity, labor, and machinery used for soil preparation, harvesting, and straw mashing. The social costs included the estimated costs of global warming (CO_2_ eq ha^−1^ × 0.0204 USD kg^−1^) [49], soil acidification (SO_2_ eq ha^−1^ × 0.62 USD kg^−1^), eutrophication (PO_4_ eq ha^−1^ × 0.71 USD kg^−1^) [27], and human health effects (0.30 × N_2_O-N + 0.20 × NO_3_-N + 3.30 × NH_3_-N) [4]. 

### 3.5. Statistical Analysis

Data were presented as means of the multiple yearly replicates. A one-way analysis of variance (ANOVA) in SAS software (ver. 6.12; SAS Institute, Cary, NC, USA) was performed to evaluate the treatment effect. Where the ANOVA was significant, treatments were compared using the least significant difference (LSD) test at a 5% level of probability. The linear–plateau and quadratic models were used to describe the relationships of grain yield or grain protein yield with N rate. The model with the highest R2 was identified as the best fit.

## 4. Conclusions

In the present study, the long-term sustainability of summer maize production through optimizing N management based on agronomic, environmental, health, and economic aspects was evaluated using the LCA and economic analysis. The main findings were: (1) The maximal mean grain yield (10.6 Mg ha^−1^) and grain protein yield (793 kg ha^−1^) were obtained under the Opt. N treatment, through better synchronizing N supply with maize N demand temporally and spatially; (2) Compared with the Con. N, lower N inputs in the Opt. N treatment significantly reduced the life-cycle pollutants, thereby substantially reducing acidification, eutrophication, global warming, and energy depletion potentials by 29%, 42%, 35%, and 18%, respectively, as well as the human health impacts by 32%; and (3) From an economic benefits perspective, the Opt. N and Opt. N*50–70% treatments achieved the highest private profitability (2038 USD ha^−1^), ecosystem economic benefit (1811 USD ha^−1^), and integrated compensation benefit (17,548 USD ha^−1^). This study shows the potential of optimizing N management, in combination with other improved management practices, to balance the trade-offs among maize productivity, environmental, and health impacts, and to further promote sustainable summer maize production on the NCP in the long term. More studies are needed to explore the extended application of optimal N management in other crops and regions, with careful consideration of the specific crop N demand and local ecological conditions.

## Figures and Tables

**Table 1:** Average costs and benefits (USD ha^−1^) of summer maize production system under five N treatments during 2008 to 2019 of the experiment.

Items	CK	Opt. N*50–70%	Opt. N	Opt. N*130–150%	Con. N
Grain yield benefit	1942 c	3195 b	3294 a	3281 ab	3267 ab
N cost	0 e	66 d	99 c	132 b	155 a
Other costs	1157 a	1157 a	1157 a	1157 a	1157 a
Private profitability	786 b	1956 a	2038 a	1992 a	1956 a
Social cost	74 e	175 d	227 c	285 b	336 a
Ecosystem economic benefit (EEB)	712 d	1797 ab	1811 a	1706 bc	1620 c
Human Capital Losses (HCL)	18 e	45 d	56 c	68 b	82 a
Integrated compensation benefit (ICB)	699 d	1754 a	1747 a	1632 b	1537 c
Social cost-private profitability ratio (%)	11.0 c	9.4 c	12.0 bc	15.5 ab	18.6 a

CK, no N; Opt. N*50–70%, 50–70% of optimal N rate; Opt. N, optimal N rate; Opt. N*130–150%, 130–150% of optimal N rate; Con. N, conventional N rate. Means followed by the same lowercase letter are not significantly different among the five N treatments at *p* < 0.05 according to LSD.

## Data Availability

The data presented in this study are available on request from the corresponding author.

## Supplementary Materials

The following supporting information can be downloaded at: https://www.mdpi.com/article/10.3390/plants12071490/s1, Figure S1: Life-cycle acidification, eutrophication, global warming, and energy depletion potential per Mg of maize grain produced under five N treatments during the years 2008–2011 and 2012–2019 of the experiment. CK, no N; Opt. N*50–70%, 50–70% of optimal N rate; Opt. N, optimal N rate; Opt. N*130–150%, 130–150% of optimal N rate; Con. N, conventional N rate. MS, agricultural materials system; FS, arable farming system. Values are means + SE. In each panel, means followed by the same letter are not significantly at *p* < 0.05 according to LSD.; Figure S2: Life-cycle acidification, eutrophication, global warming, and energy depletion potential per Mg of maize grain protein produced under five N treatments during the years 2008–2011 and 2012–2019 of the experiment. Values are means + SE. CK, no N; Opt. N*50–70%, 50–70% of optimal N rate; Opt. N, optimal N rate; Opt. N*130–150%, 130–150% of optimal N rate; Con. N, conventional N rate. MS, agricultural materials system; FS, arable farming system. In each panel, means followed by the same letter are not significantly at *p* < 0.05 according to LSD; Table S1: Changes in the agricultural inputs and optimal N management practices during 2008–2019; Table S2: N fertilizer application rate (kg N ha^−1^) under five N treatments during 2008–2019; Table S3: Factors and equations for emissions estimation from the application of fertilizer; Table S4: Two-way analysis of variance of the nitrogen (N) treatments with year (Y) interactions on tested parameters of summer maize grown with varied levels of N application under field condition. ***, ** and * indicate significant difference at *p* < 0.001, 0.01, 0.05 levels, respectively, and ns indicate no significant difference; Table S5: Average grain yield, grain protein concentration, and grain protein concentration in summer maize production system under five N treatments during 2008–2011 and 2012–2019; Table S6: The comparison of environmental impacts in maize production system in North China Plain and other countries (as determined by a literature search).
