# Supplementary material for: Optimizing Agronomic, Environmental, Health and Economic Performances in Summer Maize Production through Fertilizer Nitrogen Management Strategies
Source: Plants (Basel). 2023 Mar 29;12(7):1490. doi: 10.3390/plants12071490 (PMC10097194; doi:10.3390/plants12071490)
Supplement: Supplementary file 1 [file plants-12-01490-s001.zip › plants-2257050-SI.pdf]

Optimizing agronomic, environmental, health and economic performances in summer maize production through fertilizer nitrogen management strategies

This Supporting Information file has 9 pages including Fig. S1-S2 and Tables S1-S6.

Table S1. Changes in the agricultural inputs and optimal N management practices during 2008-2019.

Input	Unit	2008-2011	2012-2019
P_2O_5	kg ha^{-1}	45	45
K_2O	kg ha^{-1}	90	90
Density	plants ha^{-1}	75000	90000
Seeding date	/	June 10th-30th	June 5th-10th
Pesticides	kg ha^{-1}	4.1	3.8
Diesel	kg ha^{-1}	46	46
Irrigation water	m^3 ha^{-1}	863	1081
Electricity	kW h ha^{-1}	574	719
N target values			
	kg ha^{-1}	V6-10 stage, 120	V6-R1 stage, 185
	kg ha^{-1}	V10-R6 stage, 190	R1-R6 stage, 165

Table S2. N fertilizer application rate (kg N ha^{-1}) under five N treatments during 2008-2019.

	CK	Opt. N*50-70%	Opt. N	Opt. N*130-150%	Con. N
2008	0	120	240	360	250
2009	0	75	150	225	250
2010	0	74	105	137	250
2011	0	135	193	251	250
2012	0	120	172	224	250
2013	0	114	163	212	250
2014	0	122	174	226	250
2015	0	102	145	189	250
2016	0	139	199	259	250
2017	0	119	170	221	250
2018	0	74	105	137	250
2019	0	74	105	137	250
Mean N rate	0	106	160	215	250

CK, no N; Opt. N*50-70%, 50-70% of optimal N rate; Opt. N, optimal N rate; Opt. N*130-150%, 130-150% of optimal N rate; Con. N, conventional N rate.

Table S3. Factors and equations for emissions estimation from the application of fertilizer.

Emissions items	Emissions factors or equations	Required information	References
NH_3-N	N rate	$7.98 + 0.099 \times N_{rate}$	Cui et al., 2018
NO_3-N	N rate	$10.7 \times \exp(0.0060 \times N_{rate})$	Cui et al., 2018
Direct N_2O-N			
Opt. N*50-70%	N rate	0.93%	Song et al., 2018
Opt. N	N rate	0.85%	Song et al., 2018
Opt. N*130-150%	N rate	0.97%	Song et al., 2018
Con. N	N rate	1.29%	Song et al., 2018
Indirect N_2O from NH_3-N	Quantity of NH_3-N	1.00%	IPCC, 2006
Indirect N_2O from NO_3-N	Quantity of NO_3-N	0.75%	IPCC, 2006
NOx-N	Total N_2O quantity	10.00%	Brentrup et al., 2004
PO_4-P	Total P quantity	1.00%	Gaynor and Findlay, 1995

Table S4. Two-way analysis of variance of the nitrogen (N) treatments with year (Y) interactions on tested parameters of summer maize grown with varied levels of N application under field condition. ***, ** and * indicate significant difference at P<0.001, 0.01, 0.05 levels, respectively, and ns indicate no significant difference.

Source of variation	Grain yield	Grain protein yield	Acidification potential	Eutrophication potential	Global warming potential	Nergy depletion potential	Human health effects
Nitrogen (N)	***	***	***	***	***	**	***
Year (Y)	**	**	**	**	*	*	**
N×Y	*	**	*	*	*	ns	*

Table S5. Average grain yield, grain protein concentration, and grain protein concentration in summer maize production system under five N treatments during 2008-2011 and 2012-2019

N treatment	Grain yield (Mg ha^{-1})		Grain protein concentration (%)		Grain protein yield (kg ha^{-1})	
	2008-2011	2012-2019	2008-2011	2012-2019	2008-2011	2012-2019
CK	6.4 c	6.2 c	7.13 c	5.74 d	389 c	295 d
Opt. N*50-70%	8.9 ab	11.0 b	7.59 b	7.36 c	566 b	721 c
Opt. N	9.4 a	11.3 ab	8.36 a	7.94 ab	658 a	860 a
Opt. N*130-150%	8.8 b	11.5 a	8.68 a	8.09 a	639 a	874 a
Con.N	9.1 ab	11.3 ab	8.38 a	7.79 b	640 a	821 b

CK, no N; Opt. N*50-70%, 50-70% of optimal N rate; Opt. N, optimal N rate; Opt. N*130-150%, 130-150% of optimal N rate; Con. N, conventional N rate. Means in a row followed by the same letter are not significantly at P<0.05 according to LSD.

Table S6. The comparison of environmental impacts in maize production system in North China Plain and other countries (as determined by a literature search).

Country or region	N rate (kg N ha^{-1})	Grain yield (Mg ha^{-1})	Global warming potential (kg CO_2 eq Mg_{-1})	Acidification potential (kg SO_2 eq Mg_{-1})	Eutrophication potential (kg PO_4 eq Mg_{-1})	Energy depletion potential (kg PO_4 eq Mg^{-1})	References
NCP-Quzhou	160	10.6	304	6.5	2.9	2.0	Opt. N treatment in this study
USA typical Corn Belt	110-181	6.3-9.1	254-825	2.7-7.8	0.7-2.3	2.1-3.1	Kim et al. (2009)
Poland	140	8.9	850	3.0	1.4	5.6	Król-Badziak et al. (2021)
NCP-Huantai	262	7.7	394	4.9	4.0	3.8	Wang et al. (2007)
NCP-Huantai	254	7.5	1230	19.9	4.4	5.2	Liang et al. (2019)
NCP-Luancheng	181	8.7	443	13.9	4.1	3.5	Liang et al. (2018)
NCP-Quzhou	182	9.1	487	11.4	2.3	2.3	Wang et al. (2014)

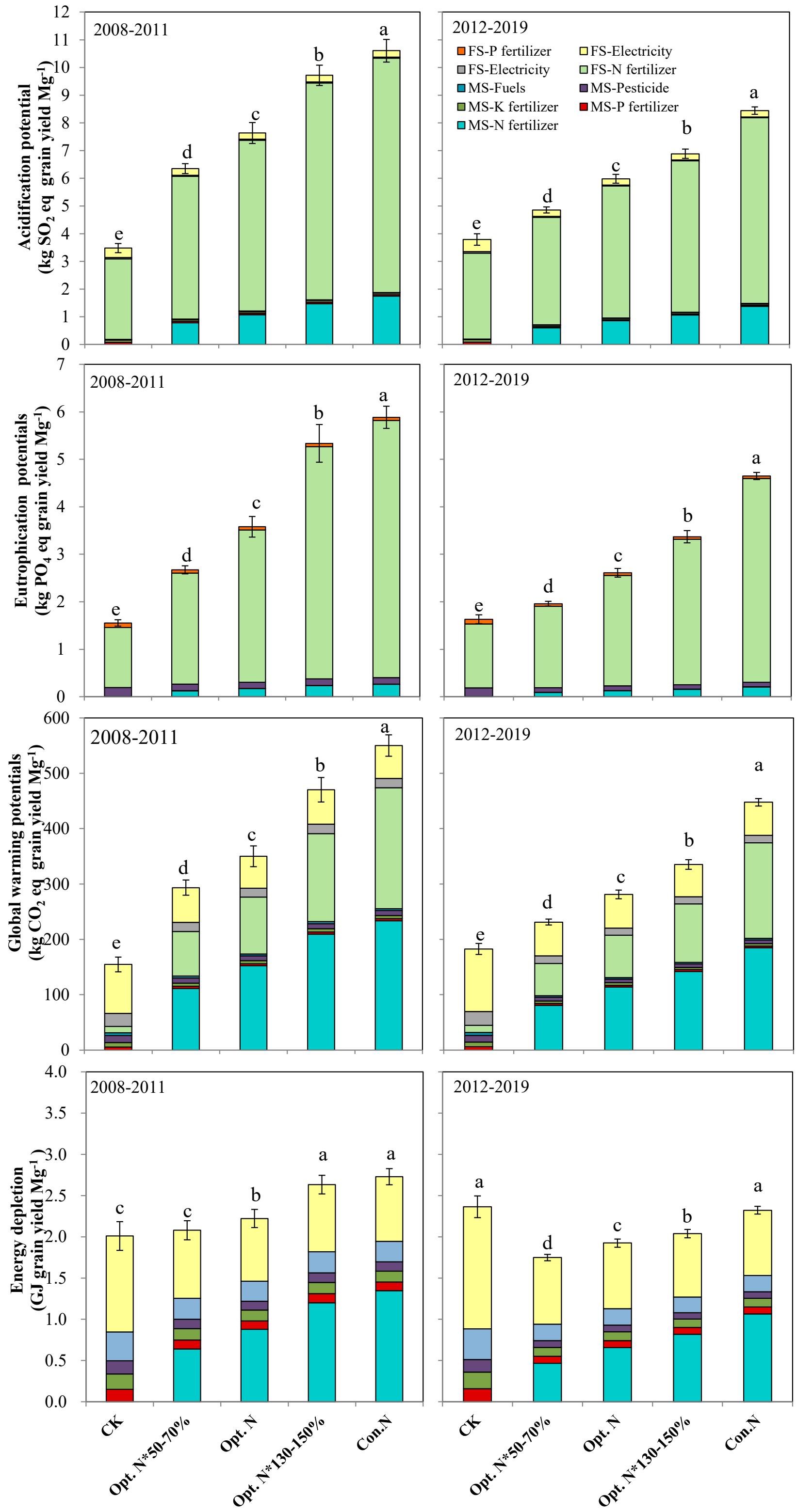

Fig. S1. Life-cycle acidification, eutrophication, global warming, and energy depletion potential per Mg of maize grain produced under five N treatments during the years 2008-2011

and 2012-2019 of the experiment. CK, no N; Opt. N*50-70%, 50-70% of optimal N rate; Opt. N, optimal N rate; Opt. N*130-150%, 130-150% of optimal N rate; Con. N, conventional N rate. MS, agricultural materials system; FS, arable farming system. Values are means + SE. In each panel, means followed by the same letter are not significantly at $P<0.05$ according to LSD.

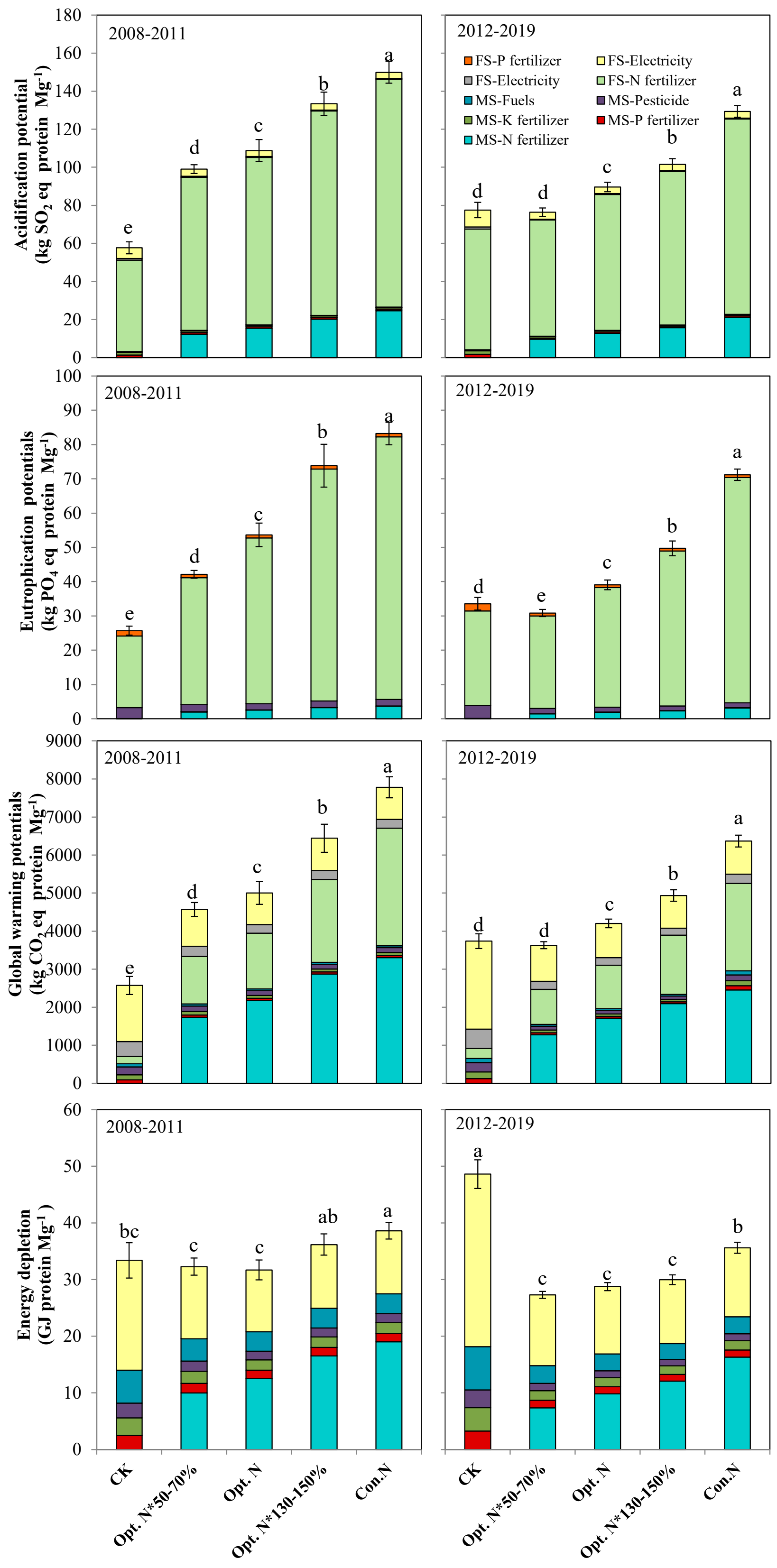

Fig. S2. Life-cycle acidification, eutrophication, global warming, and energy depletion potential per Mg of maize grain protein produced under five N treatments during the years

2008-2011 and 2012-2019 of the experiment. Values are means + SE. CK, no N; Opt. N*50-70%, 50-70% of optimal N rate; Opt. N, optimal N rate; Opt. N*130-150%, 130-150% of optimal N rate; Con. N, conventional N rate. MS, agricultural materials system; FS, arable farming system. In each panel, means followed by the same letter are not significantly at P<0.05 according to LSD.
